# Efficacy and safety of corticosteroids, hyaluronic acid, and PRP and combination therapy for knee osteoarthritis: a systematic review and network meta-analysis

**DOI:** 10.1186/s12891-023-06925-6

**Published:** 2023-11-30

**Authors:** Xiaochen Qiao, Lei Yan, Yi Feng, Xiaoyan Li, Kun Zhang, Zhi Lv, Chaojian Xu, Sen Zhao, Fengrui Liu, Xihua Yang, Zhi Tian

**Affiliations:** 1 Second Clinical Medical College, Shanxi Medical University, 382 Wuyi Road, Taiyuan, Shanxi 030001 PR China; 2 Department of Orthopedics, Shanxi Key Laboratory of Bone and Soft Tissue Injury Repair, The Second Hospital of Shanxi Medical University, 382 Wuyi Road, Taiyuan, Shanxi 030001 PR China; 3 Department of Orthopedics, JinZhong Hospital Affiliated to Shanxi Medical University, 689 Huitong South Road, Jinzhong, Shanxi 030600 PR China; 4 Xihua Yang Shanxi Province Cancer Hospital, Shanxi Hospital Affiliated to Cancer Hospital, Chinese Academy of Medical Sciences, Cancer Hospital Affiliated to Shanxi Medical University, Taiyuan, Shanxi 030013 PR China; 5 Orthopedics Department, First Hospital of Shanxi Medical University, Taiyuan, Shanxi 030001 PR China; 6 Taiyuan Hand Surgery Hospital, Taiyuan, Shanxi 030001 PR China

**Keywords:** Knee osteoarthritis, Corticosteroids, Hyaluronic acid, Platelet-rich plasma, Meta-analysis

## Abstract

**Objective:**

There are many injectable treatments for knee osteoarthritis with different characteristics and effects, the aim is to understand which one can lead to better and safer results.

**Methods:**

The PRISMA principles were followed when doing the literature search. Web of Science databases, Embase, the Cochrane Library, PubMed, and the Wanfang database were searched to identified randomized controlled trials that assessed the efficacy of corticosteroids (CSC), platelet-rich plasma (PRP), hyaluronic acid (HA), and combination therapy in treating KOA. Risk of bias was assessed using the relevant Cochrane tools (version 1.0). The outcome measure included the visual analog scale (VAS) score, the Western Ontario and McMaster Universities Osteoarthritis (WOMAC) score, and treatment-related adverse events. The network meta-analysis was performed using STATA17 software and a Bayesian stratified random effects model.

**Results:**

Network meta-analysis using the Bayesian random-effects model revealed 35 studies with 3104 participants. PRP showed the best WOMAC score at a 3-month follow-up, followed by PRP + HA, HA, placebo, and CSC; PRP + HA scored the highest VAS, followed by PRP, CSC, HA, and placebo. PRP, CSC, HA, and placebo had the highest WOMAC scores six months following treatment; PRP + HA showed the best VAS scores. PRP showed the best WOMAC score at 12 months, followed by PRP + HA, HA, placebo, and CSC; The best VAS score was obtained with PRP, followed by PRP + HA, HA, and CSC. No therapy demonstrated a rise in adverse events linked to the treatment in terms of safety.

**Conclusions:**

The current study found that PRP and PRP + HA were the most successful in improving function and alleviating pain after 3, 6, and 12 months of follow-up. CSC, HA, PRP, and combination therapy did not result in an increase in the incidence of treatment-related side events as compared to placebo.

**Supplementary Information:**

The online version contains supplementary material available at 10.1186/s12891-023-06925-6.

## Introduction

Knee osteoarthritis (KOA) is a chronic joint condition characterized by cartilage degeneration and an increase in bone growth in the knee joint [1, 2]. The knee joint’s primary symptoms include discomfort, swelling, and mobility problems. As the population ages, more people are developing KOA, which has a major impact on middle-aged and older people’s health and quality of life [3–5]. As a multifactorial disease that develops over a long period of time [6], KOA has always been a huge burden on individuals and society as a whole due to its high disability rate [7].

Currently, intra-articular injection (IAI) remains the primary element of non-surgical therapy for KOA [8]. The evidence that is now available demonstrates that this therapy can significantly reduce short-term pain for patients with KOA and improve joint function while also having a minimal risk of patient injury [9, 10]. Interestingly, botulinum toxin and ozone have also been proven to be used for injection into joints to treat KOA [11, 12]. HA, a naturally occurring glycosaminoglycan, serves as a crucial component of synovial fluid in joints, functioning as a lubricant and a shock absorber with elastic properties during joint movement [13]. In addition, A has the following functions: proteoglycan and glycosaminoglycan synthesis, anti-inflammatory, mechanical, subchondral, and analgesic actions [14]. HA is a widely used conservative treatment for OA because of both its indirect and direct analgesic effects on joints. Many clinical studies have shown that HA supplementation has a good effect on KOA, but HA may increase the risk of adverse events, such as transient pain at the injection site [15]. Knee Joint injection of CSC has a lasting effect of weeks to months [16]. The anti-inflammatory and immunosuppressive effects of corticosteroids are obvious [17], and CSC can raise the knee joint’s relative viscosity and HA concentration [18]. Regarding the intra-articular CSC’s effective duration, there is disagreement. IAI of PRP has gained widespread attention in recent years as a novel and successful alternative therapy for patients with KOA [19]. The mechanism of local injection of PRP is that it can relieve joint pain and reduce synovial hyperplasia and effusion in the joint cavity [20]. PRP is considered to have a variety of important physiological functions, such as anti-inflammation, analgesia, and promoting chondrocyte proliferation and cartilage repair. Besides, PRP can also regulate the progression of KOA by regulating WNT and IL-1 signaling [21]. In recent years, scholars have combined them to investigate the possibility of dual therapy [22]. Wang et al. discovered that individuals taking hyaluronan and corticosteroids together had pain alleviation and improved knee function faster than either medication alone. At 6 months, however, there was no discernible difference [23]. Huang et al. discovered that whereas corticosteroids and hyaluronan were equivalent in terms of pain alleviation after three months, PRP injections were superior in terms of long-term pain relief [24]. John et al.‘s study found that PRP has better efficacy than HA [13], but another study found no difference between the two [25]. Overall, there are still many controversies in this field, and there is an urgent need for an article to integrate all the evidence and provide a credible recommendation.

In this study, a Bayesian network meta-analysis of randomized controlled trials (RCTs) was conducted to evaluate the effectiveness and safety of CSC, HA, PRP, and their combination in treating KOA.

## Materials and methods

### Ethical approval

This meta-analysis did not need ethical approval since no new clinical raw data were collected or used; rather, the analysis was conducted based only on previously published research that had already been granted ethical approval.

### Literature search

In accordance with the PRISMA checklist [26], a comprehensive search was carried out in the Web of Science databases, Embase, the Cochrane Library, PubMed, and the Wanfang database to collect English publications until December 2022. The search criteria consisted of keywords such as “corticosteroids OR steroids OR hyaluronic acid OR platelet rich plasma OR PRP OR placebo (PLA)” and the condition of interest, “knee OR osteoarthritis OR KOA”. To find more pertinent literature, a manual search and literature tracking techniques were also performed. Supplemental File 1 provides details of the search strategy.

### Inclusion and exclusion criteria of literature

The following were the study’s inclusion criteria: (1) RCTs involving patients with KOA; (2) original research; (3) studies that reported at least two of the following treatments: HA, CSC, PRP, combination therapy, and/or placebo; and (4) includes VAS OR WOMAC outcome scores or the proportion of patients who had adverse effects. The following were the exclusion criteria: (1) literature review; (2) non-randomized studies; (3) failure to get original data; and (4) low-quality or duplicate publications. Two authors conducted an independent search of all references and any disagreements were resolved by a vote of all authors.

### Data extraction

Two authors (XQ and LY) conducted data extraction independently, discussed their findings, and reached an agreement in case of any disagreements. Each qualifying study’s first author, publication year, country, methods of treatment, length of time, sample size, outcome measures, and follow-up time points were all recorded.

### Methodological quality assessment

Two authors (XL and LY) independently evaluated the quality of the included literature, and a third researcher was invited to help resolve any differences. Review Manager Software5.4 (The Nordic Cochrane Collaboration, Copenhagen)’s risk of bias summary was used to examine the following biases: sequence generation, allocation concealment, blinding, incomplete outcome data, no selective outcome reporting, and other sources of bias. Each criterion was judged to have a low, unclear, or high risk of bias.

### Statistical analysis

#### Data synthesis

Stata 17.0 was used for data processing and analysis, and to draw related graphs [27]. For dichotomous variable data, we estimated the odds ratio (OR) with 95% confidence intervals (CIs), and for continuous variable data, we estimated the standardized mean differences (SMD) with 95% CIs. The initial model update iteration number was set to 10,000, and the continuous update iteration number was set to 10,000. To mitigate the impact of the starting value, the first 10,000 annealing times were utilized, and sampling began after 10,001 times. We calculated the relative ranks of the intervention groups using a consistency model and then displayed the percentages of the surface under the cumulative ranking curve (SUCRA). We conducted a network meta-analysis for each outcome only when the intervention groups could be connected to create a network; however, comparisons of support surfaces allocated to the same group were not excluded from the overall systematic review.

#### Assessing the certainty of evidence

A detailed review of the completeness of the literature search was used to estimate the possibility of publication bias. This involved creating funnel plots for each paired meta-analysis that contained more than 10 studies, as well as a network-adjusted funnel plot. Furthermore, the depth of the literature search and the amount of unpublished data acquired were considered.

## Results

### Literature search

**Fig. 1:**
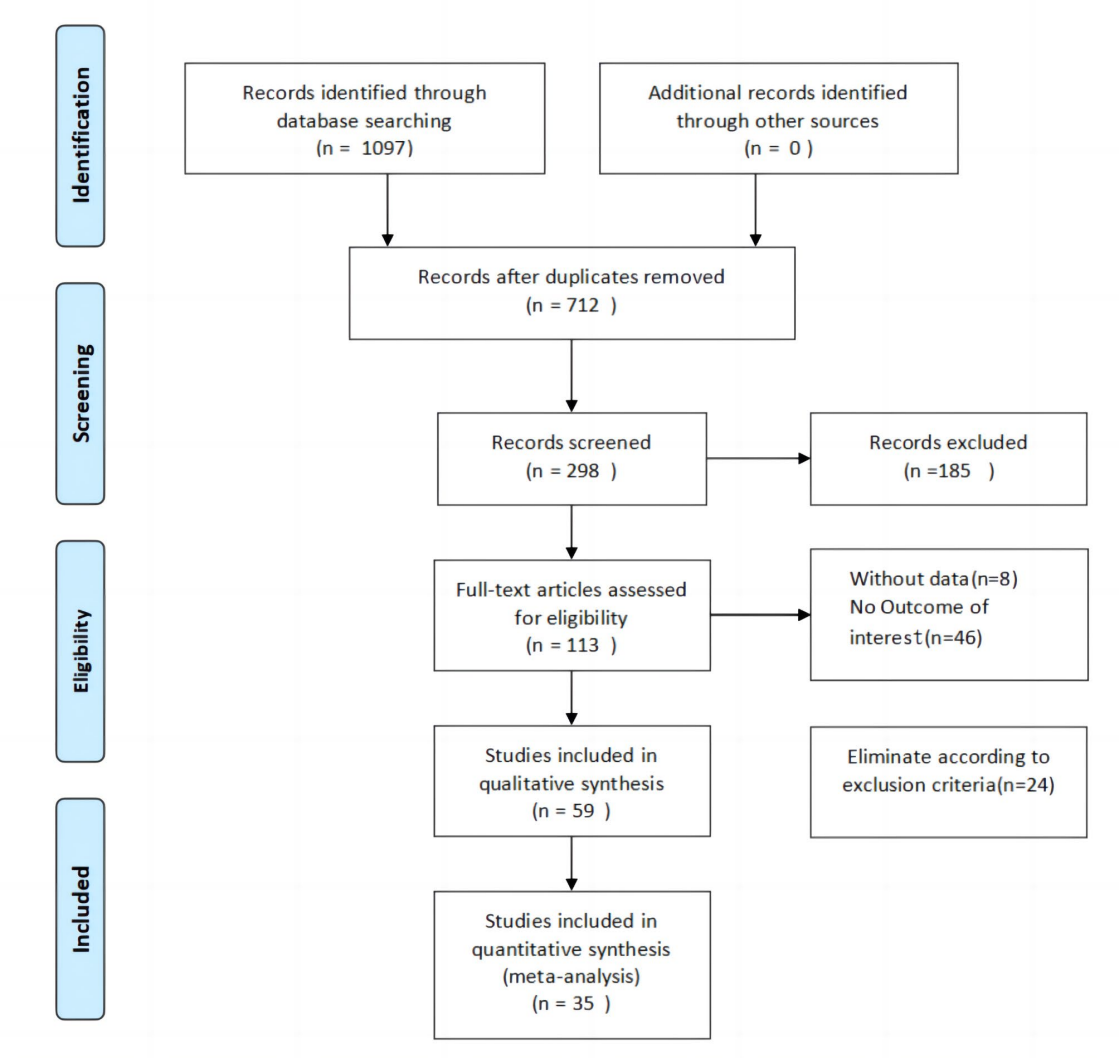
Flowchart of the study procedure

Out of 1097 RCTS pertaining to KOA identified through the database search, 1062 were eliminated for diverse reasons, including 385 duplicates, while 712 articles were screened by title and abstract, thereby resulting in the exclusion of 599 irrelevant studies. Afterward, a thorough examination of 113 articles led to the elimination of 8 articles that lacked an index of existing data, 46 articles that did not present the outcome of interest, and 24 articles that were not connected to the outcome. This ultimately brought the meta-analysis down to 35 studies. Figure 1 illustrates the particulars of the literature search.

### Patient demographics and methodological quality assessment

**Fig. 2:**
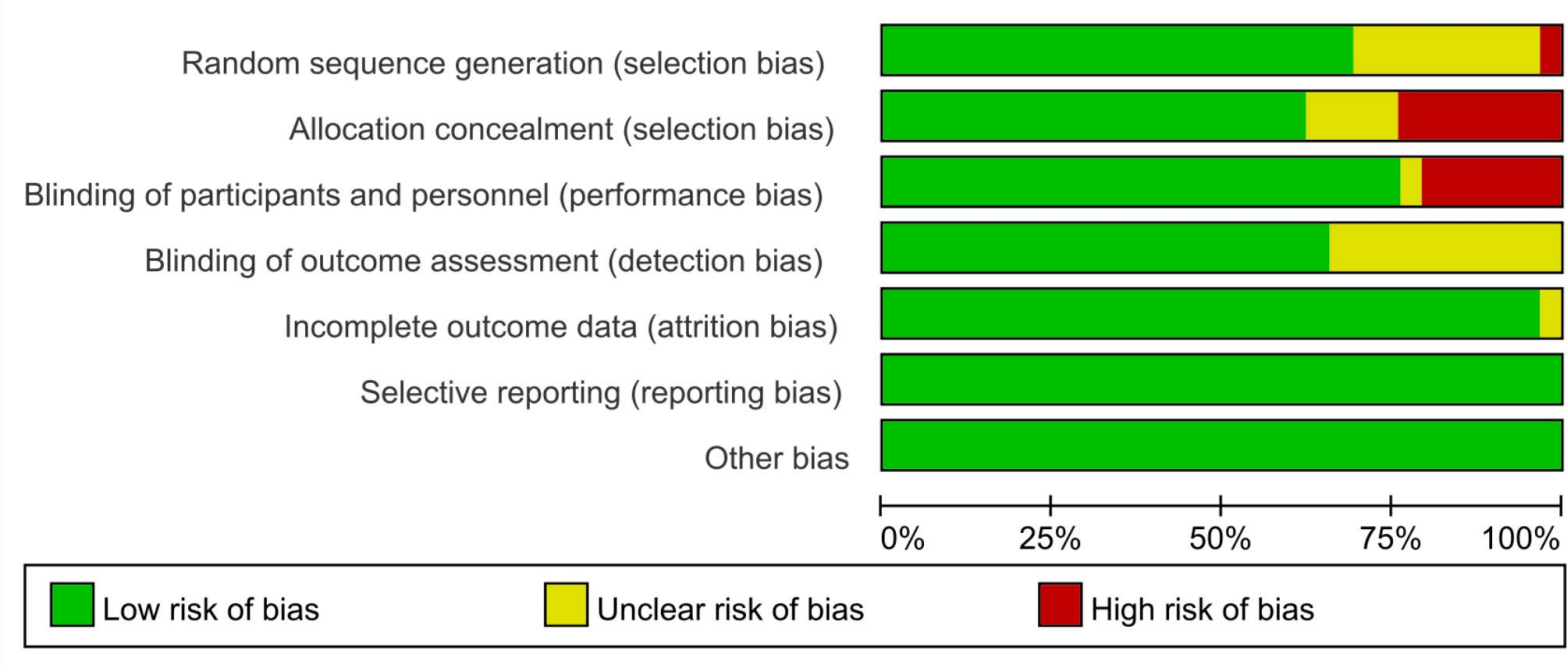
Summary of the risk of bias

Included were 35 RCTs with 3104 patients from 16 countries in total. The mean age of the enrolled patients was 59.1 years, and 61.3% of them were female. The course of treatment ranged from 3 to 24 months. Follow-up time reached 3 months in 35 studies, 6 months in 31 studies, 9 months in 14 studies, 12 months in 14 studies. Table 1 presents a comprehensive list of included studies along with their characteristics. The majority of studies utilized blinding techniques. Furthermore, the hazards of attrition, reporting, and unidentified bias are minimal. Methodological evaluations had a minimal risk of bias and were of high quality. Figure 2 depicts the methodological quality evaluation.

**Table 1:** Characteristics of 36 studies included in the meta-analysis

Study	Country	Intervention	Duration	Sample	Age,mean (SD)		Gender(male/female)		OUTCOME
					EXP	CON	EXP	CON	
Askari et al., 2016	Iran	CSC VS HA	3months	140	57.0 ± 1.9	58.5 ± 8.3	12/57	9/62	VAS
Buendía-Lópe et al., 2018	Spain	PRP VS HA	52weeks	65	56.15 ± 3.001	56.63 ± 2.9	16/17	15/17	WOMAC、VAS、Adverse effects
Cerza et al., 2012	Italy	PRP VS HA	24weeks	120	66.5 ± 11.3	66.2 ± 10.6	25/35	28/32	WOMAC
Cole et al., 2016	USA	PRP VS HA	52weeks	99	55.9 ± 10.4	56.8 ± 10.5	28/21	20/30	WOMAC、VAS
Raeissadat et al., 2021	Iran	PRP VS HA	12months	101	56.09 ± 6.0	57.91 ± 6.7	13/39	13/39	WOMAC、VAS
Park et al., 2021	Korea	PRP VS HA	6months	110	60.6 ± 8.2	62.3 ± 9.6	16/39	8/47	WOMAC、VAS、Adverse effects
Dório et al., 2021	Brazil	PRP VS PLA	24weeks	41	66.4 ± 5.6	66.1 ± 7.5	1/19	2/19	WOMAC、VAS、Adverse effects
Elik et al., 2019	Turkey	PRP VS PLA	6months	57	61.30 ± 7.91	60.19 ± 6.80	1/29	3/24	WOMAC、VAS、Adverse effects
Lana et al., 2016	Brazil	PRP VS HA VS PRP + HA	12months	105	PRP: 60.9 ± 7,HA:60 ± 6.6,PRP + HA:62 ± 6.1		PRP: 29/7,HA:33/3,PRP + HA:27/6		VAS
Xu et al., 2020	China	PRP VS HA VS PRP + HA	24months	122	PRP:56.9 ± 4.2,HA:57.1 ± 3.4,PRP + HA:57.9 ± 4.1		PRP: 10/20,HA:5/15,PRP + HA:8/20		Adverse effects
Sun et al., 2021	China	PRP VS PRP + HA	6months	85	60.6 ± 8.4	58.4 ± 8.1	18/21	22/17	WOMAC 、VAS
Yu et al., 2018	China	PRP VS HA VS PRP + HA VS PLA	52weeks	360	PRP: 46.2 ± 8.6,HA:51.5 ± 9.3,PRP + HA:46.5 ± 7.5,PLA:56.2 ± 8.4		PRP:50/54,HA:48/40,PRP + HA:50/46,PLA:42/30		WOMAC、Adverse effects
Elksniņš-Finogejevs et al., 2020	Latvia	PRP VS CSC	12months	40	66.4 ± 8.4	70.2 ± 9.2	3/17	5/15	VAS、Adverse effects
Yan et al., 2020	China	HA VS PLA	26weeks	440	61.5 ± 7.9	61.6 ± 7.8	50/170	48/172	WOMAC、Adverse effects
Petterson et al., 2018	USA	HA VS PLA	26weeks	369	59.5 ± 8.0	58.7 ± 9.2	75/109	79/106	WOMAC、VAS、Adverse effects
Huang et al., 2019	China	PRP VS HA VS CSC	12months	120	PRP:54.5 ± 1.2,HA:54.8 ± 1.1,CSC:54.3 ± 1.4		PRP:19/21,HA:21/19,CSC:35/15		WOMAC、VAS、Adverse effects
Kesiktas et al., 2020	Turkey	PRP VS HA	3months	36	52.7 ± 8.3	55.1 ± 10.3	4/14	2/16	WOMAC、VAS
Wang et al., 2021	China	CSC + HA VS HA	3months	57	61.7 ± 15.3	59.2 ± 13.8	12/16	12/17	Adverse effects
Davalillo et al., 2015	Mexico	HA VS CSC	12months	200	62.7 ± 0.6	62.8 ± 0.6	59/38	57/41	WOMAC、Adverse effects
Martino et al., 2018	Italy	PRP VS HA	24months	192	52.7 ± 13.2	57.5 ± 11.7	53/32	47/35	VAS
Duymus et al., 2015	Turkey	PRP VS HA	12months	102	60.4 ± 5.1	60.3 ± 9.1	1/32	1/33	WOMAC、VAS
Elsawy et al., 2017	Egypt	HA VS CSC	6months	60	52.5 ± 12.5	50.2 ± 11.4	18/42		WOMAC、VAS
GÜVENDİ et al., 2018	Turkey	CSC VS PRP	6months	57	62.8 ± 1.7	62.3 ± 1.6	2/15	1/18	WOMAC
Ismaiel et al., 2019	Egypt	CSC VS PRP	6months	92	61.1 ± 11.6	62.9 ± 11.6	9/31	23/29	VAS
Jubert et al., 2017	Spain	CSC VS PRP	6months	65	68 ± 7.17	65.56 ± 8.6	6/24	6/23	VAS
Khongwir et al., 2018	India	HA VS CSC	6months	45	70.8 ± 4.82	71.2 ± 5.22	—	—	WOMAC
Lin et al., 2019	China	PRP VS HA VS PLA	12months	87	PRP: 61.17 ± 13.08,HA:62.53 ± 9.9, PLA:62.2 ± 11.71		PRP: 9/22,HA:10/19, PLA:10/17		WOMAC
Louis et al.,2018	France	PRP VS HA	3months	54	53.2 ± 11.7	48.5 ± 11.5	14/10	11/13	WOMAC、VAS、Adverse effects
McAlindon et al.,2017	USA	CSC VS PLA	24months	140	59.1 ± 8.3	57.2 ± 7.6	33/37	38/32	Adverse effects
Naderi et al.,2018	Iran	PRP VS CSC	6months	77	58.55 ± 8.79	59.09 ± 7.79	7/27	5/28	VAS
Patel et al., 2013	India	PRP VS PLA	6months	78	53.11 ± 11.55	53.65 ± 8.17	11/16	6/17	VAS、Adverse effects
Spakova et al., 2012	Slovakia	PRP VS HA	6months	120	52.80 ± 12.43	53.20 ± 14.53	33/27	31/29	WOMAC、Adverse effects
Su et al., 2018	China	PRP VS HA	18months	86	54.16 ± 6.56	53.13 ± 6.41	11/14	12/18	WOMAC、VAS、Adverse effects
Tammachote et al., 2016	Thailand	HA VS CSC	6months	99	62.6	61	7/43	13/46	WOMAC、VAS
Wang et al., 2022	China	PRP VS HA	6months	110	61.87 ± 5.46	63.00 ± 5.33	12/42	16/40	WOMAC

CSC: corticosteroids;HA: yaluronic acid ; PRP:latelet-rich plasma ; PLA: Placebo ; WOMAC:Western Ontario and McMaster Universities Osteoarthritis; VAS:visual analogue scale

### WOMAC scores

At the 3-month follow-up, 1319 patients were included in the study, with 15 reported WOMAC scores. The PRP groups performed the best in terms of the outcomes (SMD=-8.79; 95% CI-15.69~-1.89), followed by PRP + HA (SUCRA value, 61.2; mean rank, 2.6); HA (SUCRA value, 48.9; mean rank, 3); PLA (SUCRA value, 38.2; mean rank, 3.5); and CSC (SUCRA value, 17.3; mean rank, 4.3).

**Fig. 3:**
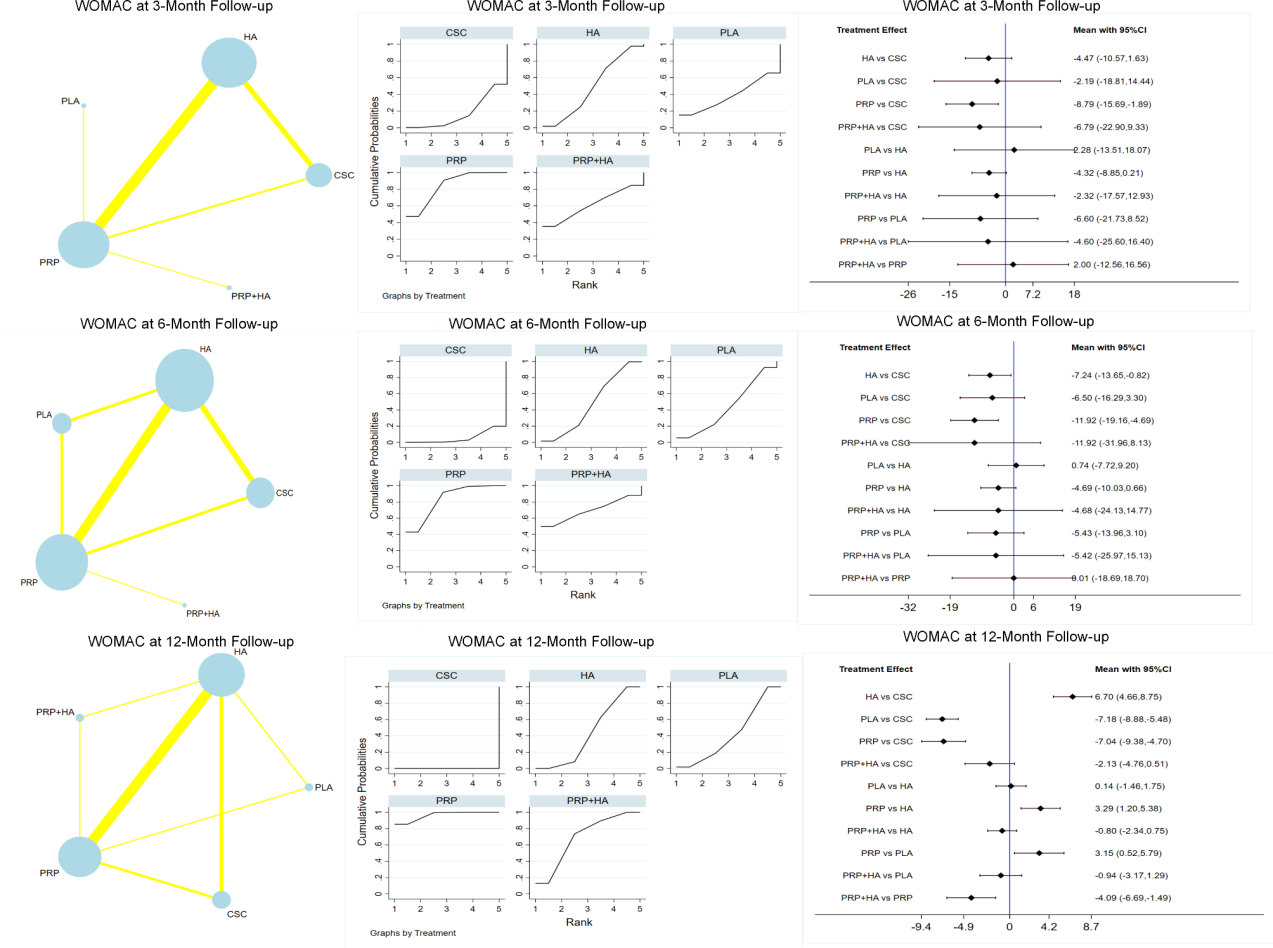
Overall network comparisons using WOMAC scores

Twenty reported WOMAC scores at 6 months of follow-up, including a total of 2310 patients, the best outcomes were shown in the PRP groups (SMD=-11.92; 95% CI: -19.16~-4.69), which were followed by PRP + HA (SUCRA value, 64.2; mean rank, 2.4), HA (SUCRA value, 50.2; mean rank, 3.0), PLA (SUCRA value, 39.9; mean rank, 3.4), and CSC (SUCRA value, 6.7; mean rank, 4.7). Ten reported WOMAC scores at 12 months of follow-up, including a total of 1148 patients, the PRP groups performed the best (SMD=-7.04;95% CI: -9.38~-4.70), followed by PRP + HA (SUCRA value, 69.0; mean rank 2.2), HA (SUCRA value, 42.8; mean rank, 3.3), PLA (SUCRA value, 42.0; mean rank, 3.3), CSC (SUCRA value, 0.0; mean rank, 5.0). Table 2; Fig. 3 provide summaries of the network meta-analysis findings. No discrepancy between the direct and indirect effects of any intervention was observed as per the nodal analysis of the intervention measures (P > 0.05). Figure 3 compares the results based on the WOMAC scores at the 3, 6, and 12-month follow-ups.

**Table 2:** Network meta-analysis treatment ranking results for each of WOMAC scores, VAS scores and adverse effects

Treatment	WOMAC scores						VAS scores						Adverse effects	
	SURCA (3month)	Mean Rank	SURCA (6month)	Mean Rank	SURCA (12month)	Mean Rank	SURCA (3month)	Mean Rank	SURCA (6month)	Mean Rank	SURCA (12month)	Mean Rank	SURCA	Mean Rank
CSC	17.3	4.3	6.7	4.7	0.0	5.0	48.2	3.1	56.7	2.7	23.3	3.3	64.2	2.8
HA	48.9	3.0	50.2	3.0	42.8	3.3	47.4	3.1	48.0	3.1	27.5	3.2	43.2	3.8
PLA	38.2	3.5	39.9	3.4	42.0	3.3	26.3	3.9	12.7	4.5			81.8	1.9
PRP	84.4	1.6	88.9	1.4	96.2	1.2	57.6	2.7	50.7	3.0	85.5	1.4	22.6	4.9
PRP + HA	61.2	2.6	64.2	2.4	69.0	2.2	70.5	2.2	81.8	1.7	63.7	2.1	81.2	1.9
CSC + HA													7.0	5.7

Note: CSC :corticosteroids; HA: hyaluronic acid ; PRP: platelet-rich plasma; PLA: placebo

### VAS scores

**Fig. 4:**
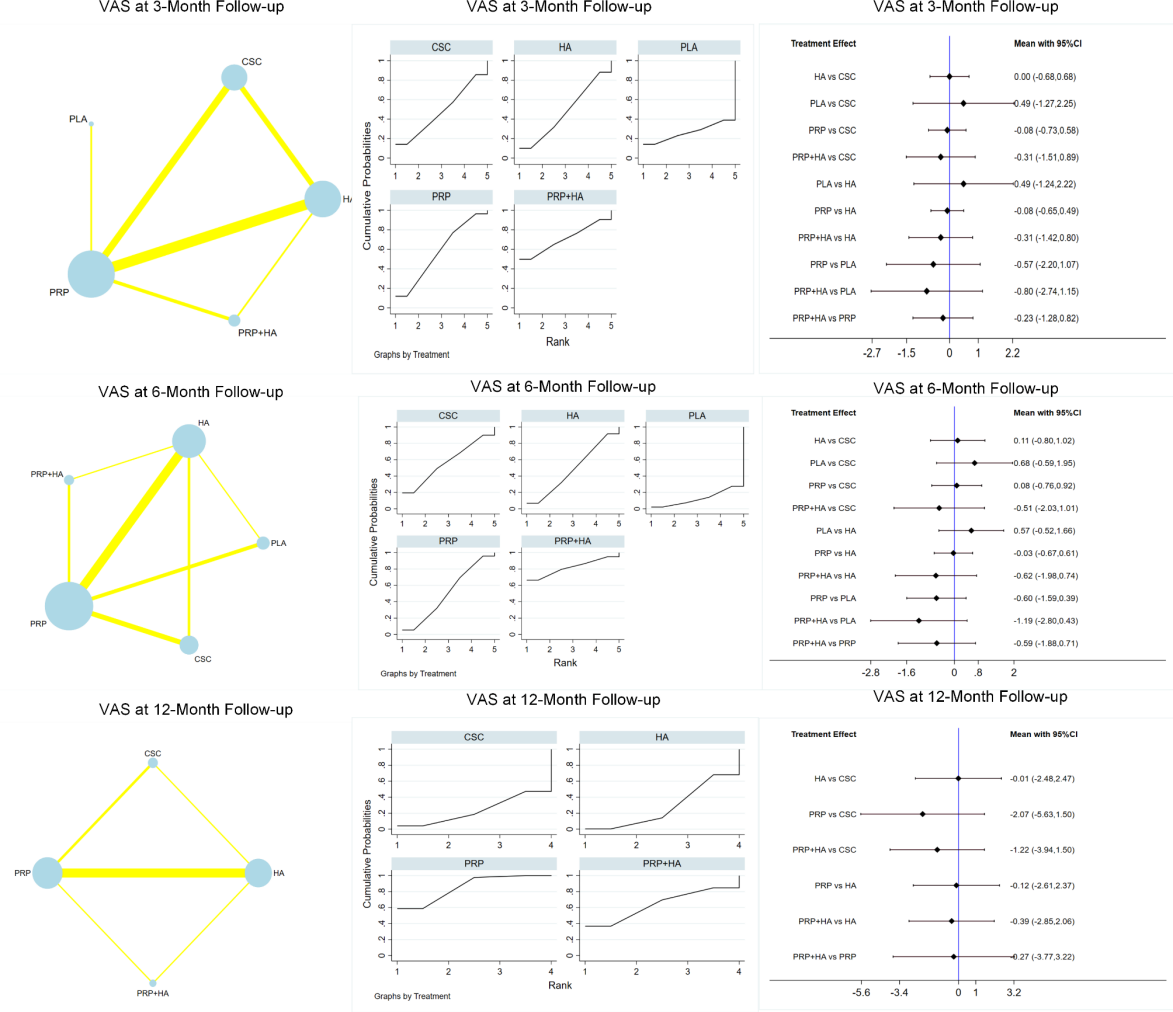
Overall network comparisons using VAS scores

In total, 1099 patients reported 15 VAS scores after 3 months of follow-up. PRP + HA had the best outcomes, with a SUCRA value of 70.5 and a mean rank of 2.2, followed by PRP (SUCRA value, 57.6; mean rank, 2.7), CSC (SUCRA value, 48.2; mean rank, 3.1), HA (SUCRA value, 47.4; mean rank, 3.1), and PLA (SUCRA value, 26.3; mean rank, 3.9). Eighteen reported VAS scores at 6 months of follow-up, including a total of 1732 patients, the PRP + HA groups showed the best outcomes (SUCRA value, 81.8; mean rank 1.7), followed by CSC (SUCRA value 56.7; mean rank, 2.7), PRP (SUCRA value, 50.7; mean rank, 3.0), HA (SUCRA value, 48.0; mean rank, 3.1), PLA (SUCRA value, 12.7; mean rank, 4.5). At the 12-month follow-up, a total of 656 patients reported 8 VAS scores, with the PRP + HA groups displaying the most favorable outcomes (SUCRA value, 85.5; mean rank, 1.4), followed by PRP + HA (SUCRA value, 63.7; mean rank, 2.1), HA (SUCRA value, 27.5; mean rank, 3.2), and CSC (SUCRA value, 23.3; mean rank, 3.3). The results of the network meta-analysis are summarized in Table 2; Fig. 4. No discrepancy between the direct and indirect effects of any intervention was observed as per the nodal analysis of the intervention measures (P > 0.05). Figure 4 compares the results based on the VAS scores at the 3, 6, and 12-month follow-ups.

### Safety

**Fig. 5:**
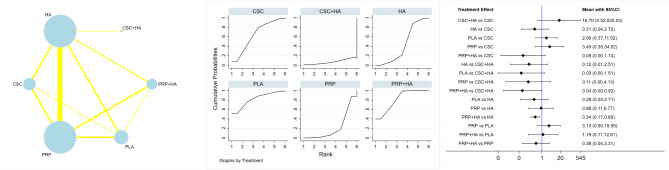
Overall network comparisons using adverse effects

Among the 2576 patients with reported adverse effects, the PRP groups demonstrated the most favorable outcomes with a SUCRA value (81.8) and a mean rank (1.9), followed by PRP + HA (SUCRA value, 81.2; mean rank, 1.9), CSC (SUCRA value, 64.2; mean rank, 2.8), HA (SUCRA value, 27.5; mean rank, 3.2), PRP (SUCRA value, 22.6; mean rank, 4.9), and CSC + HA (SUCRA value, 7.0; mean rank 5.7). The results of the network meta-analysis are summarized in Table 2; Fig. 5. No discrepancy between the direct and indirect effects of any intervention was observed as per the nodal analysis of the intervention measures (P > 0.05). Figure 5 showed a comparison of results based on adverse effects.

## Discussion

After 3, 6, and 12 months of follow-up, the Bayesian network meta-analysis revealed that PRP and PRP + HA IAIs were superior to CSC, HA, and placebo in alleviating pain and improving joint function. However, no discernible changes between CSC, HA, and placebo were found. Regarding safety, the incidence of adverse events associated with the other interventions was not significantly higher than that of the placebo.

According to this study, PRP proved to be superior to PRP + HA, CSC, HA, and PLA in enhancing joint function. Additionally, PRP + HA was found to be better than PRP, CSC, HA, and PLA in reducing pain. The incidence of adverse events did not significantly increase with other interventions, as compared to placebo. According to a prior network meta-analysis, the PRP group was more effective than CSC, HA, and placebo [28]. In the research by Zhao and his colleagues, the PRP + HA scheme was shown to be more effective than PRP alone in alleviating knee pain and raising the WOMAC overall score [29]. Compared with lower-molecular-weight hyaluronic acid, the highest-molecular-weight hyaluronic acid may be more efficacious in treating knee OA [30]. However, viscosupplementation is associated with an increased risk for serious adverse events [31]. Another study showed that intraarticular CS is more effective on pain relief than intraarticular HA in short term (up to 1 month), while HA is more effective in long term (up to 6 months) [32]. Autologous blood can be subjected to centrifugation to extract PRP, which can increase the platelet concentration by nearly ten times [33]. Upon activation, it exhibits the ability to discharge macrophages and growth factors, consequently promoting the elimination of necrotic tissue, reducing the inflammatory reaction, and facilitating the repair and regeneration of articular cartilage [34, 35]. HA, an essential element of synovial fluid and articular cartilage [10], is a polysaccharide with a high molecular weight. Injecting HA into the knee joint cavity can physically lubricate the joint surface, reduce erosion, biologically nourish the articular cartilage, and stimulate the production of endogenous HA, thereby delaying the onset of additional joint disease [36, 37]. Besides, HA has also been proven effective in obese individuals [38]. According to Marmotti et al. [39], the incorporation of HA into PRP has been found to significantly enhance the growth of chondrocytes and enhance cartilage regeneration capabilities. PRP and HA have been shown in studies to synergistically increase the functioning of signaling molecules such as inflammatory molecules, catabolic enzymes [40], cytokines, and growth factors, thus contributing to the successful treatment of KOA [41].

Some studies are consistent with the results of this study [42, 43], which found that CSC and HA showed similar results compared to placebo. However, there are also other studies that have reached different conclusions [30, 44, 45], finding that CSC and HA are more effective than placebo. The study demonstrated that the analgesic efficacy of the two therapies varied with time. Particularly, the VAS score of the intra-articular CSC group was considerably lower than that of the intra-articular HA group after 1 month, suggesting that CSC had a higher short-term analgesic impact than HA. However, in the long run, HA exhibited a greater analgesic effect than CSC [32]. No significant difference in pain relief was found between HA and placebo(saline)by Colen et al. [46]. According to a meta-analysis, intra-articular corticosteroid injection is an effective treatment for pain relief with no increase in treatment-related adverse reactions when compared to placebo [47]. Najm et al. discovered that CSC decreased pain and increased function early after administration (≤ 6 weeks) compared to placebo. However, there were no clinical improvements when compared to HA [10]. Based on our analysis, the only treatments that clinically showed improvement in both cases were PRP and PRP + HA. The effectiveness of CCS and HA is uncertain. Although treating KOA with PRP and HA combination may be more expensive and difficult, it may still be a preferable option to the expenses and risks of surgery. Nevertheless, there is still a shortage of cost-effectiveness studies that examine the combination of PRP and HA for KOA treatment, as well as studies that investigate PRP or HA alone, indicating a need for further research.

There are several limitations to this study: First of all, the main limiting factor is the lack of available data between the included studies. Secondly, some authors conducted a single injection, whereas others performed repeated injections. Thirdly, the duration of treatment and follow-up was diverse. Fourthly, we only included studies written in English, which may result in the loss of some research data. Lastly, the use of different formulations in different studies of HA may lead to bias.

## Conclusions

The study’s SUCRA value backs the application of PRP and PRP + HA for appropriate patients with KOA. PRP is likely the most effective pain-relieving treatment with the lowest incidence of adverse effects, followed by PRP + HA. The differences in treatment effects were minor and might not have any significant impact on clinical outcomes.

### Electronic supplementary material

Below is the link to the electronic supplementary material.

Supplementary Material 1

## Data Availability

All data generated or analyzed during this study are included in this published article [and its supplementary information files].
