# Supplementary material for: Efficacy and safety of corticosteroids, hyaluronic acid, and PRP and combination therapy for knee osteoarthritis: a systematic review and network meta-analysis
Source: BMC Musculoskelet Disord. 2023 Nov 30;24:926. doi: 10.1186/s12891-023-06925-6 (PMC10687893; doi:10.1186/s12891-023-06925-6)
Supplement: Supplementary file 1 — Supplementary Material 1 [file 12891_2023_6925_MOESM1_ESM.doc]

**Supplementary file 1. Search strategy**

**Pubmed**

((corticosteroids) OR (hyaluronic acid ) OR (platelet-rich plasma) OR (CS) OR (HA) OR (PRP)) AND ((controlled clinical trial) OR (randomized controlled trial)) AND ((knee osteoarthritis) OR (KOA) OR (knee OA) OR (OA) OR (osteoarthritis) )

**Web of Science**

(TS=(corticosteroids) OR TS=(hyaluronic acid) OR TS=(platelet-rich plasma) OR TS=(CS) OR TS=(HA) OR TS=(PRP)) AND ( TS=(controlled clinical trial) OR TS=(randomized controlled trial)) AND (TS=(knee osteoarthritis) OR TS=(KOA) OR TS=(knee OA) OR TS=(OA) OR OR TS= (osteoarthritis) )

**Embase**

#1 'corticosteroids'/exp OR 'hyaluronic acid'/exp OR 'platelet-rich plasma'/exp OR 'CS'/exp OR 'HA'/exp OR 'PRP'/exp

#2 'controlled clinical trial'/exp OR 'randomized controlled trial'/exp

#3'knee osteoarthritis'/exp OR 'KOA'/exp OR 'knee OA'/exp OR 'OA'/exp OR 'osteoarthritis'/exp

#4 #1 AND #2 AND #3

**The Cochrane Library**

#1 (corticosteroids) OR (hyaluronic acid ) OR (platelet-rich plasma) OR (CS) OR (HA) OR (PRP)

#2 (controlled clinical trial) OR (randomized controlled trial)

#3(knee osteoarthritis) OR (KOA) OR (knee OA) OR (OA) OR (osteoarthritis)

#4 #1 AND #2 AND #3
